# Bibliography

**Published:** 1893-08

**Authors:** 


### Bibliography.

FORMULAIRE PRATIQUE POUR LES MALADIES DE LA BOUCHE ET DES DENTS. Par G. Viau, Professeur à l'École Dentaire de Paris, Société d'Éditions scientifiques. Paris, 1893.

This book is somewhat similar to the "American Compend" in that the author endeavors to condense the various pathological conditions the dentist is called upon to treat into as small a compass as possible. He then follows this with various formulæ suitable for the treatment. These are prepared with care, and, doubtless, in many cases will be of advantage. They are generally selected from writers in various parts of the world, hence have a special value in that they are the result of wide experience and different climatic conditions. The formulæ for dentifrices are quite extended, covering twenty pages, principally from the French and German.

The second part is devoted entirely to cocaine anæsthesia and an addendum upon the agent recently introduced,—tropacocaine.

COLUMBIAN EDITION. LETTERS FROM A MOTHER TO A MOTHER ON THE CARE OF CHILDREN'S TEETH. By "Mrs. M. W. J." Third Edition. Published by the Wilmington Dental Manufacturing Company. Philadelphia, 1893.

This well-known, and in many respects very valuable, little book has reached its third edition, giving evidence of filling a place in the instruction of those for whom it was intended.

The necessity of this instruction must be apparent to every practising dentist. The real work of saving teeth begins or should begin in the nursery, where it is probably the least regarded. The suffering which might be saved by timely knowledge and care cannot be estimated. Some of the ideas sought to be inculcated under the chapter on "Food Principles" are not in accord with recent teachings, especially that in regard to the use of lime-water in increasing the density of hard tissues. There is no evidence that lime taken into the system in this way is assimilated.

The book is written in a way well adapted to arrest attention, and could it be freely distributed outside of professional circles it would do an incalculable amount of good to generations yet to come.

